# Multiple Spinal Tuberculosis with Severe Kyphosis: A Case Report

**DOI:** 10.3389/fsurg.2022.815514

**Published:** 2022-04-01

**Authors:** Liyi Chen, Chong Liu, Zhen Ye, Tuo Liang, Shengsheng Huang, Jiarui Chen, Tianyou Chen, Hao Li, Wuhua Chen, Xuhua Sun, Ming Yi, Jie Jiang, Hao Guo, Xinli Zhan

**Affiliations:** First Affiliated Hospital, Guangxi Medical University, Nanning, China

**Keywords:** spinal tuberculosis, abscess, debridement, orthopedic, kyphosis

## Abstract

**Background:**

The purpose of this study was to analyze the clinical efficacy of a patient with multiple tuberculosis of the spine combined with severe kyphosis.

**Case Summary:**

A 56-year-old male patient presented with low back pain with numbness and fatigue in both lower extremities for 5 months. Chest and back showed intermittent acid pain. The patient had not a history of constitutional symptoms. Preoperative X-ray and CT examination revealed multiple vertebral segmental bone destruction, multiple abscess calcification, and severe kyphosis. Preoperative MRI examination showed that the tuberculous abscess broke through the spinal canal and compressed the spinal cord and nerve roots. The patient underwent posterior lumbar abscess debridement, expanded decompression of the spinal canal, and nerve lysis in our hospital. The operation time was 70 min, and the intraoperative blood loss was 200 ml. The postoperative drainage volume was 250 ml. The patient was hospitalized for a total of 13 days, and the patient’s vital signs were stable before and after surgery. The patient was satisfied with the treatment.

**Conclusion:**

For the patient with multiple spinal tuberculosis complicated with severe kyphosis and multiple calcified abscesses in this study, we considered performing abscess debridement to relieve the symptoms of back pain and achieved good clinical efficacy.

## Introduction

According to the Global Tuberculosis Report 2020, global TB in China accounted for 8.4% (1). Spinal tuberculosis is the most common form of extrapulmonary tuberculosis (2). Although patients with spinal tuberculosis usually have chest and back pain (3), scoliosis is also common (4, 5). Scoliosis due to spinal tuberculosis is usually associated with cumulative sagittal and coronal plane imbalance (4). Thoracolumbar kyphosis could be surgically treated with a 66% corrective effect (6). The effect of sagittal correction was still the same after a long follow-up (7). However, for patients with severe scoliosis, the incidence of the failure rate of internal fixation was 7.15% (8). Whether patients with severe kyphosis caused by spinal tuberculosis should be treated with orthopedic surgery is still worthy of further consideration.

Although abscess debridement and orthopedic surgery for patients with spinal tuberculosis combined with scoliosis had been reported in the literature, the kyphosis angle of tuberculous scoliosis was not more than 100° (9). In this study, we reported for the first time a patient with multiple spinal tuberculosis complicated with severe kyphosis and multiple calcified abscesses underwent abscess debridement.

## Case Presentation

A 56-year-old male patient presented with low back pain with numbness and fatigue in both lower extremities for 5 months. Chest and back showed intermittent acid pain. The patient had radiating pain in both lower extremities. The patient’s visual analog scale (VAS) for back pain was as high as 9 points. The Oswestry disability index (ODI) of patient quality of life was 78 points. The patient’s neurological status was diagnosed as grade C on the American Spinal Injury Association (ASIA) scale. The patient had not a history of constitutional symptoms, such as loss of appetite, night sweats, fever, or weight loss. In addition, the patient had no history of pulmonary tuberculosis and AIDS. The patient’s spinal tuberculosis was initially diagnosed through clinical symptoms, imaging examinations and anti-tuberculosis treatment. The patient agreed to be admitted for further treatment to undergo surgery. Patient was routinely prescribed anti-tuberculosis therapy with drugs including isoniazid, rifampicin, ethambutol, and pyrazinamide for 3–4 weeks prior to surgery. The patient underwent electrocardiogram and echocardiography to assess cardiac function, and lung function was assessed according to lung CT and pulmonary function tests.

### Physical Check

The physiological curvature of the cervical spine was straightened. Thoracic vertebra presents kyphosis deformity, with the 10th thoracic spinous process as the apex of backward deformity, local tenderness, and local percussion pain. The physiological curvature of the lumbar spine disappeared and the lumbar spine presented kyphosis deformity, with tenderness and percussion pain in lumbar 2,3,4 spinous processes. The patient had no sensory impairment. The muscle strength of the left lower limb was grade III, and the muscle strength of the right lower limb was normal. There was no abnormality in the examination of extremities muscle tension. The right knee reflex is hyperactive and the left is normal. The left Achilles tendon reflex was weakened and the right side was normal. Tomas’s sign was positive. Hoffmann sign was negative, as well as Babinsky sign, Chaddock sign, Oppenheim sign, Gordon sign, patellar clonus, and condyle clonus.

### Laboratory Examination

A blood test was performed after admission (**Table 1**). Blood routine: white blood cells 5.84*10^9^/L, hemoglobin 145.8 g/L, platelet count 244.9*10^9^/L. Inflammatory markers were examined, C-reactive protein <10.00 mg/L, erythrocyte sedimentation rate 8 mm. Liver function examination showed aspartate aminotransferase 48 U/L, alanine aminotransferase 47 U/L, albumin 42.1 g/L. Renal function examination showed urea 5.66 mmol/L, creatinine 73 µmol/L, and uric acid 294 µmol/L. Tumor markers test results showed CA199 18.08 U/ml, CA193 19.75 U/ml, CA125 9.87 U/ml, AFP 1.68 ng/ml and CEA 1.5 ng/ml.

**Table 1:** A blood test was performed in our hospital.

Blood project	Measured value
White blood cells (*10^9^/L)	5.84
Hemoglobin (g/L)	145.80
Platelet (*10^9^/L)	244.90
C-reactive protein (mg/L)	<10
Erythrocyte sedimentation rate (mm)	8.00
Aspartate aminotransferase (U/L)	48.00
Alanine aminotransferase (U/L)	47.00
Albumin (g/L)	42.10
Urea (mmol/L)	5.66
Creatinine (µmol/L)	73.00
Uric acid (µmol/L)	294.00
CA199 (U/ml)	18.08
CA193 (U/ml)	19.75
CA125 (U/ml)	9.87
AFP (ng/ml)	1.68
CEA (ng/ml)	1.50

### Imaging Examination

**Figure 1:**
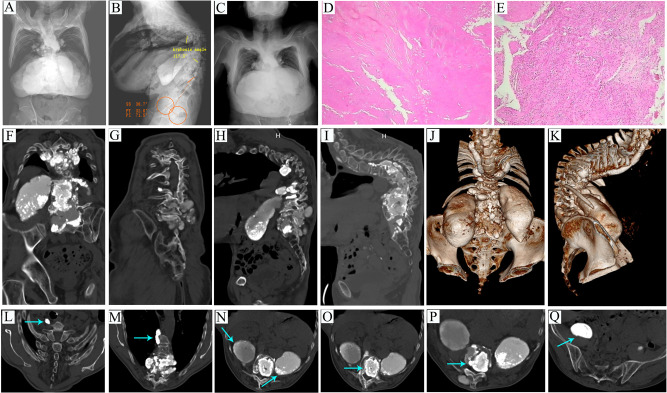
(**A–C**) Preoperative X-ray examination. (**A**) Coronal Cobb angle cannot be measured due to interference with multiple calcified abscesses. (**B**) The kyphosis angle was measured at 117.3° in the sagittal plane. Pelvic parameters were measured as follows, SS 38.7°, PT32.8°, PI71.5°. (**C**) Chest radiographs showed no obvious lung abnormalities. (**D** and **E**) Postoperative pathological examination. (**D**) This image shows caseous necrosis. (**E**) This image shows inflammatory fibrous tissue. (**F–Q**) Preoperative CT examination. (**F**), Multiple calcified abscesses in the coronal plane. (**G**) Bone destruction in the coronal plane. (**H**) Multiple calcified abscesses in the sagittal plane. (**I**) Bone destruction in the sagittal plane. (**J**) CT 3D reconstruction in the coronal plane. (**K**) CT 3D reconstruction in the sagittal plane. (**L**) The arrow points to a cervical paravertebral calcified abscess in the cross-section. (**M**) The arrow points to a thoracic paraspinal calcified abscess in the cross-section. (**N**) The arrow points to bilateral psoas calcified abscess in the cross-section. (**O**) The arrow points to the vertebral calcified abscess in the cross-section. (**P**) The arrow points to the intervertebral calcified abscess in the cross-section. (**Q**) The arrow points to the pelvic calcified abscess in the cross-section.

**Figure 2:**
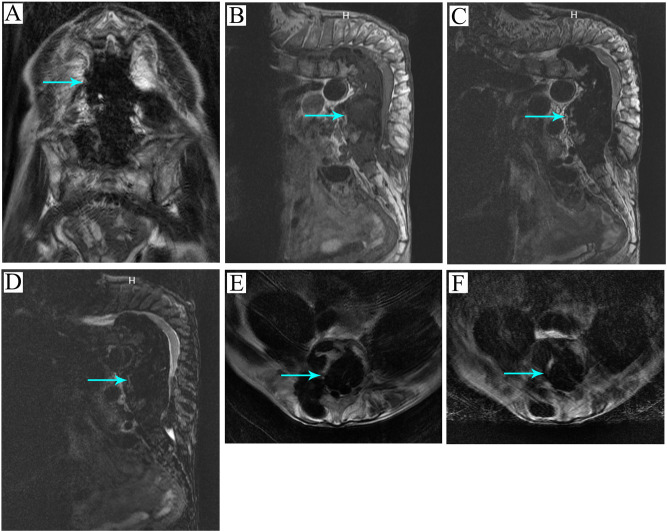
Preoperative MRI examination. (**A**) The arrow points to the calcified abscess in the T1 coronal sequence. (**B**) The arrow points to the calcified abscess in the T1 sagittal sequence. (**C**) The arrow points to the calcified abscess in the T2 sagittal sequence. (**D**) The arrow points to the calcified abscess in the T2 lipid pressing sagittal sequence. (**E**) The arrow points to the calcified abscess in the T2 lipid pressing cross-section sequence. (**F**) The arrow points to the calcified abscess in the T2 cross-section sequence.

All the imaging data of the patients were taken in our hospital. Preoperative Cobb angle could not be measured from the X-ray coronal sequence due to interference with calcified abscesses (Figure 1A). The kyphosis angle was measured at 117.3° from the X-ray sagittal sequence (Figure 1B). Pelvic parameters were measured as follows, SS 38.7°, PT32.8°, PI71.5° (Figure 1B). Chest radiographs showed no obvious lung abnormalities (Figure 1C). Pathological examination results showed caseous necrosis and inflammatory fibrous tissue (Figures 1D,**E**). Combined with clinical data, it was consistent with spinal tuberculosis. CT scan revealed calcification of the abscess and destruction of the vertebral body in various locations (Figures 1F–**Q**). MRI showed many calcified abscesses in the sagittal plane, coronal plane, and cross-section (Figure 2).

### Surgical Procedures

After successful anesthesia, the patient was placed in the prone position, with routine skin disinfection and towel laying. The posterior median longitudinal incision was made with the lumbar spinous process as the center. The skin, subcutaneous fascia, and ligaments were cut layer by layer, The paravertebral muscles were removed along the spinous process and the lamina. A hemilamina retractor was placed to retract the muscles. After full exposure of the operating field, the spinous process of the lumbar 2–3 vertebral body and simultaneous lumbar 2–3 level laminectomy was performed. The ligament flavum and the dura mater were incised to expose the caseous abscess in the spinal canal, and the caseous necrotic tissue was completely removed. Spinal cord compression and nerve root adhesion were relieved during the operation. After the wound was thoroughly rinsed and no active bleeding was detected, streptomycin powder was placed around the lesion. After placing the drainage tube, the incision was sutured layer by layer, and the operation was finished.

## Results

**Figure 3:**
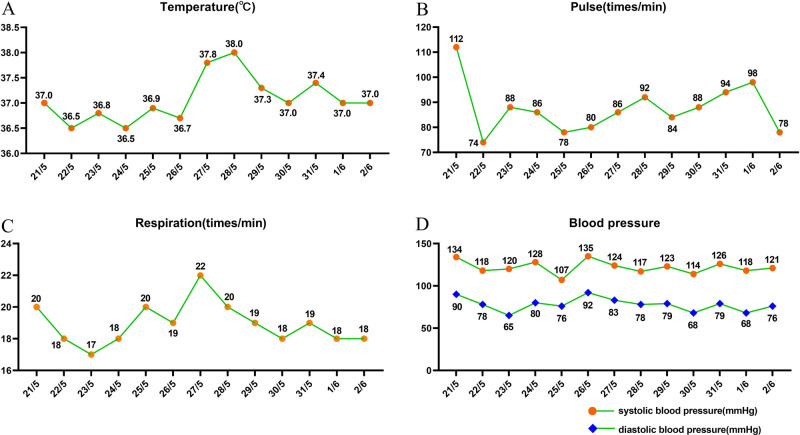
Patient’s vital signs were measured daily in the hospital. (**A**) The results of temperature examination. (**B**) The results of pulse examination. (**C**) The results of respiration examination. (**D**) The results of blood pressure examination.

The patient underwent posterior lumbar abscess debridement, expanded decompression of the spinal canal, and nerve lysis under general anesthesia in our hospital. Patients and their families refuse to perform internal fixation implantation reconstruction orthopaedics because they could not afford huge economic and surgical risks. The operation time was 70 min, and the intraoperative blood loss was 200 ml. The patient returned to the ward after surgery. The postoperative drainage volume was 250 ml. There was no redness and swelling in the incision of the back, and no obvious flushing and swelling of the surrounding skin. The patient continued anti-tuberculosis treatment postoperatively, including isoniazid, rifampicin, ethambutol, and pyrazinamide. During postoperative follow-up, the patient’s condition improved and anti-tuberculosis treatment was effective. Multiple drug resistance was not observed. The patient was hospitalized for a total of 13 days, and the patient’s vital signs were stable before and after surgery (Figure 3). The patient was discharged from the hospital 8 days after surgery with no obvious low back pain, and the numbness and fatigue of both lower limbs were significantly relieved compared with that before surgery. The patient was satisfied with the treatment. We did not find any recurrence of spinal tuberculosis in our follow-up for up to 2 years after surgery. The patients were satisfied with the treatment effect.

## Discussion

Tuberculosis placed a huge burden on developing countries (1). Spinal tuberculosis usually presents as back pain, but in severe cases, it also caused neurological impairment (5, 10). In this case, treatment of the patient usually required surgical intervention (11). Anterior or posterior approach treatment of lumbar tuberculosis could achieve satisfaction. In this study, the patient’s severe kyphosis was considered unsuitable for a long operation. We considered posterior approach surgery to reduce operative time and intraoperative blood loss, which was consistent with the advantages of posterior approach surgery reported in the literature (11).

Spinal tuberculosis destroyed the vertebrae severely and was usually accompanied by kyphosis, which caused the severe imbalance of the spine in the sagittal plane (12). The kyphosis deformity angle reached 47.7° and could be treated surgically (12). Although Alexander et al. studied 42 patients with spinal tuberculosis and kyphosis who underwent surgical treatment for kyphosis improvement, the mean kyphosis angle did not exceed 50° (49.2° ± 14.3° in average) (13). However, when the kyphosis angle was more than 80°, long-term follow-up is needed to determine whether surgery could improve sagittal plane balance and lung function (14). Although Wong et al.’s 34-year follow-up study recommended early surgical intervention, Frankel grades worsened in 3 patients and did not improve in 5 patients after surgery in 16 patients (15). In this study, the kyphosis angle of the patient was as high as 117.3°, and due to patient refusal of kyphosis orthopaedic surgery, we did not perform orthopedic surgery. For patients with severe kyphosis (kyphosis angle 92.5°), the outcome of orthopedic surgery treatment was satisfactory after halo traction for 3–7 weeks (16). In this study, the patient’s tuberculosis foci compressed the spinal cord and nerves, requiring timely surgery to relieve the compression. Halo traction was not available, and the patient did not agree to perform orthopedic surgery due to economic considerations. we did not consider orthopedic surgery, which was consistent with the treatment methods reported before (17).

In addition to the symptoms of tuberculosis poisoning, the common manifestation of spinal tuberculosis was a paraspinal abscess, which damaged the vertebral body and aggravated kyphosis deformity, and even damaged the neurological function (18). Abscess debridement and nerve decompression was an effective treatment for patients^,^ back pain (19). Recent literature had reported that abscess drainage could also achieve good clinical outcomes (20, 21). In this paper, the patient had multiple abscess calcification, so drainage was not suitable for this case. Routine abscess debridement surgery achieved the same clinical effect (11). The case of bilateral calcified psoas abscess was rarely reported in the literature (17). However, bilateral calcified psoas abscess combined with multiple calcified abscesses in the cervical spine, thoracic spine, lumbar spine, and the pelvic cavity was reported for the first time in this paper.

The patient underwent posterior lumbar abscess debridement, expanded decompression of the spinal canal, and nerve lysis in our hospital. The operation time was short and the amount of blood loss was less. Postoperative vital signs were stable and there were no operative complications. This paper had reported for the first time a case of multiple spinal tuberculosis with severe kyphosis and multiple calcified abscesses involving the cervical spine, thoracic spine, lumbar spine, bilateral psoas major muscle, and pelvic cavity. The patient was satisfied with the treatment.

However, there were some limitations in the article. The article was a single case report, and the treatment effect need to be verified by multiple cases.

## Conclusion

For the patient with multiple spinal tuberculosis complicated with severe kyphosis and multiple calcified abscesses in this study, we considered performing abscess debridement to relieve the symptoms of back pain and achieved good clinical efficacy. Whether orthopedic surgery was necessary for such a patient should be fully considered.

## Data Availability

The raw data supporting the conclusions of this article will be made available by the authors, without undue reservation.
